# A statewide initiative to improve healthcare transition from pediatric to adult care for individuals with neurodevelopmental disabilities: Lessons learned from engaging stakeholders

**DOI:** 10.1016/j.hctj.2025.100115

**Published:** 2025-08-06

**Authors:** Susan Shanske, Lindsay MacAuley, Jamara Possemato, Lindsay Hunt, Tuba Rashid Khan, Sarah Spence

**Affiliations:** a BRIDGES Adult Transition Program, Boston Children’s Hospital, USA; b Department of Neurology, Boston Children’s Hospital, USA; c Center for Primary Care, Harvard Medical School, USA; d Department of Neurology (retired), Boston Children’s Hospital, USA

**Keywords:** Neurodevelopmental disabilities, Healthcare transition, Quality improvement, Partnerships, Statewide initiatives

## Abstract

**Background:**

Transition from pediatric to adult healthcare is challenging for patients with Neurodevelopmental Disabilities (NDD). Patients, families, and providers endure systemic barriers. Our objective was convening stakeholders in Massachusetts to identify specific challenges and target solutions for improvement.

**Methods:**

This initiative involved a multistep stakeholder engagement process to improve healthcare transition for individuals with NDD. A steering committee (28 participants) and four task forces (72 participants overall) focused on major improvement themes (e.g., workforce development, practice infrastructure, clinical care, and financing). These groups included multidisciplinary providers, payers, advocacy organizations, state agencies and family partners and met routinely over 18-months to identify barriers and solutions, creating resources to share with key stakeholders. A statewide summit was convened to disseminate results (n = 256 participants). Quality improvement approaches guided initiative efforts and evaluation. Evaluation included interviews with adult providers caring for young adults with NDD, surveys of family members on their satisfaction with the transition process, and surveys of steering committee/taskforce members on their satisfaction with the process and recommendations for future change.

**Results:**

During the 18-month period, the steering committee and taskforce participants created resources and developed and refined a driver diagram that built a foundation for future improvement work. Provider interviews described a lack of adult providers with NDD expertise as a barrier, while availability of care coordination and ability to accommodation patient/family needs were key facilitators. Family surveys indicated low confidence for successful transition prior to transfer to adult providers, but high satisfaction with process once transfer had occurred. Summit participants reported better understanding of healthcare transition needs of the NDD population and an ability to set related goals. When asked to identify change ideas generated at the summit, participants contributed over 100 suggestions, ranging from clinic-level interventions to system-level improvements, reflecting enthusiasm for this work. The majority reported feeling satisfied with the initiative’s process and that it was a good use of time.

**Conclusion:**

This model succeeded in building relationships, developing resources collaboratively and identifying goals and improvements to address healthcare transition for those with NDD. This initiative could serve as a roadmap for convening successful transition improvement efforts.

## 1 Introduction

Transition to adulthood is a challenging time, especially for adolescents and young adults with neurodevelopmental disabilities (NDD). While education law has explicitly outlined the need for formal transition planning for students with NDD for many years,^1^ attempts to formalize transition planning in the healthcare realm, while rapidly expanding, are less well established. This is especially challenging as patients with congenital and pediatric acquired illnesses are living longer into adulthood, including those with complex conditions that include NDD.^2^, ^3^ There is also a higher prevalence of autism diagnoses in recent years.^4^, ^5^ Since these patients typically have a normal lifespan, this growing population will require transition services, including quality healthcare which addresses their unique needs.

The consensus statement from the American Academy of Pediatrics (AAP), the American Academy of Family Physicians (AAFP) and the American College of Physicians (ACP)^6^ specifically identifies several “special populations”, including individuals with developmental and/or intellectual disabilities, as facing additional challenges with the transition to adult healthcare. These include considerations around decision making capacity, and lack of accessibility and accommodations to address diverse physical challenges, sensory needs, communication and cognitive deficits to best prepare individuals with NDD for engagement in transition. Further, for those with medical complexity, leaving the familiarity of well-known teams, routines, and physical environment is a challenge. Many people with NDD have comorbid behavioral health conditions, for which there are a very limited number of treating providers available. Lastly, social complexity such as lack of family and community support and financial insecurity combine with other challenges and co-occurring conditions, worsening transition outcomes.

Young adults with NDD require additional supports during the transition period given their unique vulnerabilities. Studies in the literature highlight some specific areas of concern, including lack of available adult providers with expertise in caring for individuals with NDD and insufficient care coordination resources to facilitate healthcare system navigation.^7^ As a result, individuals with NDD may lack access to care and experience poorer health-related outcomes, including more limited physical activity, higher rates of smoking^8^, lower rates of cancer screening^9^, challenges with mental health^10^, and even earlier age of death^11^. With increased focus on adults with disabilities as a population of unrecognized health disparities^12^, more attention is required at the pivotal transition of pediatric to adult healthcare for people with NDD.

To address these important issues, we founded an initiative engaging multi-disciplinary stakeholders in our home state: the Massachusetts Initiative to Improve Healthcare Transition for Individuals with Neurodevelopmental Disabilities. Here, we describe the various steps entailed in the first 2 years of this initiative - from starting with a small workgroup of practitioners through our growth to a large entity including a steering committee and taskforces and culminating in a statewide day long summit that shared our learnings. The effort included different levels of convening appropriate stakeholders to engage in shared problem-solving activities. This manuscript describes the initial phase of a multi-step initiative regarding the foundational convening efforts; and it can serve as a roadmap for others to follow in this critical work by sharing state-level findings that could guide other states or regions.

## 2 Methods

### 2.1 Process of initiative creation

#### 2.1.1 Moving from a workgroup to a structured, statewide initiative

In 2018, a small group of providers at a quaternary pediatric free standing teaching hospital in the state of Massachusetts (USA) created a workgroup to address transition efforts for adolescents and young adults with NDD. Specifically, developmental behavioral pediatricians, child neurologists and other pediatric specialty providers were seeking care for their adult patients and had few, if any, adult colleagues to whom they could refer. Pediatric providers individually networked and created some partnerships, but the number of adult patients still seen in the pediatric setting remained high. There was general acknowledgement that the systemic problems facing youth during transition could not be addressed only with additional preparation on the pediatric side. Broad and deep engagement with stakeholders in all aspects of the transition process and experience was required. Thus began a multi-year commitment to address transition for youth with NDD. The purpose, as defined in the initiative charter by consensus of the initiative leadership, was to “facilitate a powerful collaboration with passionate stakeholders to identify best practices for improving the transition to adult healthcare for individuals with NDD”. The decision was made to look beyond just a few local institutions, recruiting participants with the ability to impact healthcare practice and policy for individuals with NDD across Massachusetts. Focus on the state level was also chosen given differences in payment policies and process in Medicaid by state.

Operational definitions were developed by consensus of the initiative leadership team to clearly outline the scope of the initiative. NDD was broadly defined to include those with intellectual disability, Autism Spectrum Disorder (ASD) and other developmental disabilities, including those related to medical conditions. Over the course of multiple meetings and discussions, the workgroup came to consensus on four key themes to be addressed ranging from the need to expand and educate the work force to navigating systemic and payment issues. The initiative was co-chaired by two members of the workgroup with content matter expertise: a pediatric neurologist with research and clinical expertise with patients with NDD and a clinical social worker who is a leader of the hospital’s larger transition efforts. Philanthropic funding supported administrative resources and QI consultation for the initiative.

#### 2.1.2 Specific steps taken in convening and engaging appropriate stakeholders

Realizing that we were limited to primarily the pediatric provider perspective during the workgroup phase, we sought to engage other key stakeholders for their insights. We targeted key perspectives that were inadequately represented in the initial workgroup. Specifically, it would be imperative to understand what adult providers and practices need to ensure successful transition and transfer, an area not well understood in clinical practice nor as well developed in the literature. Secondly, ensuring a patient and family voice in the entire process was prioritized as critical. Efforts were made to fully engage these particular perspectives through recruitment of family members to each group.

STEP 1 (9 months): A small grant from the medical school was used to focus on understanding successful models in adult practices. We performed standardized interviews with a small number of what were considered successful practices who were well known for caring for adults with NDD as identified by provider reputation. We wanted to determine knowledge and educational needs and any systems and infrastructure change needs. The concept was to learn what is required to provide care and what might be needed to scale up the successful approaches. Over a 9 month period one workgroup member performed nine in person interviews, notes from which were transcribed for later review. Both primary care and specialty care providers (behavioral/cognitive neurologists and neuropsychologists) were included to determine essential elements of comprehensive, integrated care management required by this population in order to understand what is needed for appropriate care. The interview process was iterative and we used snowball sampling, identifying some informants ourselves and then further informants from those we interviewed. Questions included: Describe your model and how it works? What finances, staff support do you need? Who are the members of the team? What is the scope – number clients/patients? What other care sources are used by your clients/patients and how do you coordinate with those? How do information and data flow? Walk through what happens with a new patient/client.

STEP 2 (2 months): To gather patient and family perspective, advocacy agencies were engaged as key stakeholders. One agency made outreach via email survey to caregivers of transition age individuals with NDD in order to gather some baseline understanding of the current state of family experience of transition. This was used to inform their involvement as representatives in the initiative, both on the steering committee and on taskforces. Questions were asked about caregiver confidence as they approached transfer of providers using 5-point Likert scale, whether or not primary and specialty care had been established (yes/no), and several questions about satisfaction with new providers and practices following transfer using a 5-point Likert scale (see Table 1; additional information regarding survey can be requested from the authors). The agency representatives were able to share the anonymous survey information as a snapshot of caregiver experience, in addition to many other lived experiences that are part of their work of the agency.

**Table 1:** a: Satisfaction with Transition to Primary Care. b: Satisfaction with Transition to Specialty Care.

	Strongly Disagree	Somewhat Disagree	Neutral	Somewhat Agree	Strongly Agree
“We were satisfied with the transition process.”	7.4 % (n = 2)	14.8 % (n = 4)	14.8 % (n = 4)	37.0 % (n = 10)	25.9 % (n = 7)
"We feel there is a good fit between the patient and the primary care provider.”	7.1 % (n = 2)	7.1 % (n = 2)	17.9 % (n = 5)	35.7 % (n = 10)	32.1 % (n = 9)
"We feel all primary care medical needs are taken care of."	10.3 % (n = 3)	6.9 % (n = 2)	10.3 % (n = 3)	41.4 % (n = 12)	31.0 % (n = 9)
"We are satisfied with the support staff at the provider’s office (i.e. helpful and responsive to questions, return phone calls, etc.).”	14.3 % (n = 4)	3.6 % (n = 1)	7.1 % (n = 2)	39.3 % (n = 11)	35.7 % (n = 10)

	Strongly Disagree	Somewhat Disagree	Neutral	Somewhat Agree	Strongly Agree
“We were satisfied with the transition process.”	10.0 % (n = 2)	20.0 % (n = 4)	30.0 % (n = 6)	35.0 % (n = 7)	5.0 % (n = 1)
"We feel there is a good fit between the patient and the specialty care provider.”	22.2 % (n = 4)	5.6 % (n = 1)	16.7 % (n = 3)	27.8 % (n = 5)	27.8 % (n = 5)
"We feel all primary care medical needs are taken care of."	22.2 % (n = 4)	5.6 % (n = 1)	11.1 % (n = 2)	44.4 % (n = 8)	16.7 % (n = 3)
"We are satisfied with the support staff at the provider’s office (i.e. helpful and responsive to questions, return phone calls, etc.).”	27.8 % (n = 5)	16.7 % (n = 3)	0.0 % (n = 0)	27.8 % (n = 5)	27.8 % (n = 5)

STEP 3 (18 months; includes STEP 2): To convene the range of identified perspectives we determined a statewide summit would be the ideal mechanism to engage stakeholders and share ideas. However, rather than have a meeting immediately just to discuss barriers which are well outlined in the literature and in the clinical experience of the providers involved, the group decided to delay the summit. This allowed time to identify best practices, discover current working models, and delve deeply into the needs of both clinicians and patients and their families. This occurred over an 18 month period.

First, we convened a steering committee to oversee and provide specific expertise in all aspects of the project. Initiative leadership used snowball outreach approach over the course of several months. We reached out to colleagues and leaders in the state referred through workgroup members. An introduction to the effort was sent via email and a follow up phone meeting was requested. During the meeting, full details of the initiative were shared to allow stakeholders to decide if they could commit to participating. Recommendations for other stakeholders to include were also solicited, to maximize grassroots networking. The steering committee included 28 people with diverse perspectives from various geographical areas of the state. There was representation from state agencies, insurers, patient research/advocacy groups, pediatric and adult clinicians from academic hospitals and community health centers and importantly families of those with NDD. Of note many of the members actually had multiple connections to the work such as a clinician with a child or sibling with NDD, an educator who was a clinician and researcher, and a state agency leader who is also a parent. The steering committee met monthly, providing guidance to the initiative and important connections in outreaching and engaging others, especially in building the taskforces.

Next, we formed four taskforces to address the themes identified by the initial workgroup, key stakeholder interviews and the steering committee as major barriers to transition efforts for this population. These included: the need for workforce development among the adult providers (*Workforce Development)*; the need for building clinical infrastructure in the adult practices (*Practice Management and Clinical Infrastructure)*; the need to optimize clinical care of this vulnerable population by identifying best practices (*Optimizing Clinical Care)*; and finally the need to improve funding mechanisms by identifying correct billing practices and working with payers (*Financing the Solution)*. Taskforce membership and leadership were chosen by recommendations from the steering committee and personal relationships among the original work group. Recruitment for taskforces utilized the same snowball networking approach described above for the steering committee. To encourage accessible participation, monthly meetings were established as virtual (using the Zoom platform) from the start of the initiative, allowing participation from stakeholders from across the state, including direct family representation. Meetings were not recorded, but written summaries were kept in a shared drive.

With guidance from the steering committee and the taskforces, initiative leaders then outlined a plan to hold a statewide summit to share our learnings. The “NDD Transition Summit” was scheduled for November 2020, with plans to engage the taskforces, invite other stakeholders into the discussion and present some solutions to the well described problems. A robust day of education, resource sharing and networking was planned. To encourage provider participation CME was offered for physicians, nurses, social workers, psychologists, etc.

### 2.2 Assessing need and building capacity

**Fig. 1:**
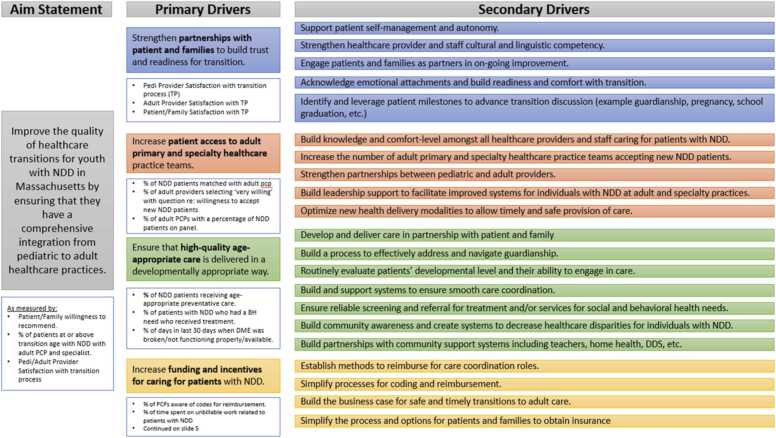
Driver Diagram.

The initiative was developed and run with a quality improvement approach guided by an experienced Quality Improvement Coach. We began by drafting the following aim to guide the work: *Improve the quality of healthcare transitions for individuals with Neurodevelopmental Disabilities (NDD) in our state by ensuring that they have a comprehensive integration from pediatric to adult healthcare practices by November 2021.* Initiative leadership and the taskforce leads met in person to identify the primary drivers necessary to accomplish our aim. Ideas from this meeting became the basis of the driver diagram (see Fig. 1) which was subsequently discussed, edited, and expanded by members of the steering committee and the taskforces. The working driver diagram gave a common language to the initiative and helped organize thinking around taskforce efforts. These all set the stage for subsequent quality improvement efforts in later phases of the initiative.

### 2.3 Task force process and objectives

The taskforces determined their own approaches to achieving their goals and deliverables. Some groups completed methodical reviews of available resources, and others brainstormed to create compilations or develop additional resources. To keep taskforces focused and productive, they were asked to create deliverables by the time of the summit. The Workforce Development taskforce had 19 participants with a goal to identify strategies and make recommendations to increase the number of trained providers and support staff confident and competent in providing care to individuals with NDD across the lifespan. The Optimizing Clinical Care taskforce consisted of 24 participants whose goal was the development of clinical pathways and practice guidelines to facilitate high quality care for adults with NDD, ensuring that all healthcare providers throughout the state know what to look for and how to care for this vulnerable population. The Practice Management and Clinical Infrastructure taskforce had 17 participants. They employed a patient/family centered approach, with a goal to develop best practice guidelines/tools to ensure optimal transition/transfer of care to adult services for patients with NDD. They aimed to improve the process for clinicians/staff in both pediatric and adult care by providing structure to integrate patients safely and effectively into adult practices that could be replicated across the state. The Financing the Solutions taskforce, with 12 participants, dealt with finance, insurance and reimbursement barriers faced by both providers and patients in the process of healthcare transition for patients with NDD. Their goal was to evaluate current practices and models and to identify strategies for incentivizing providers and adult healthcare systems to take on patients with NDD.

### 2.4 Summit structure and objectives

In November 2020 the NDD summit offered an opportunity to convene involved and interested stakeholders. Members of the taskforces, the steering committee as well as other interested parties across the state joined to share change ideas and inspire progress. Topics were proposed by steering committee and taskforce members and the agenda was developed by initiative leadership with review and approval of the steering committee and taskforces. There were three objectives of the summit:

1. Identify models for best practice in pediatric to adult healthcare transition for individuals with NDD in the state.
2. Describe quality improvement strategies to implement changes in practice.
3. Engage in solution-focused collaboration with stakeholders across the state.

Attendance was incentivized by providing continuing education credit for participants. Original plans for an in-person summit changed due to the COVID 19 pandemic and it was held virtually. To achieve the goal of shared learning, the first keynote address featuring a national expert on transition for individuals with NDD. Next came a perspectives panel, moderated by a leader from the state Department of Public Health, which included an adult provider, a pediatric provider, a parent, and a patient. Participants then heard presentations from the four taskforces who discussed their work and shared their deliverables via didactic presentation. Resources including taskforce deliverables were posted on our institution’s continuing education website, with plans to maintain the library of materials through this platform to create a community of interested stakeholders. In addition to the taskforce deliverables, a call for poster presenters yielded 18 posters featuring various efforts and lessons learned that were disseminated at the summit. A major feature of the day was an interactive component using a “world cafe” exercise, a roundtable brainstorming method where participants share ideas in one small group then move to another group to build on further idea sharing.^13^ This was accomplished using break out rooms on the virtual platform. Participants utilized the driver diagram that had been developed and further considered change ideas. In the afternoon, participants attended breakout sessions that went more deeply into details of some of the suggested interventions (see Table 2). To close the day, the co-chairs summarized the initiative output, including suggestions made at the summit itself, and challenged all participants to go home and utilize a QI approach and test out one change immediately following the summit.

**Table 2:** Break Out Sessions.

**BREAKOUT SESSION 1**	**BREAKOUT SESSION 2**
*Providers and Families Co-Creating Medical Summaries to Aid in Transition*	*Developing a Directory of Adult Providers: Facilitating Access*
*Integrating Information on Healthcare Needs of Transition Age Youth with NDD into Training Programs*	*Operation House Call: Impacting Advocacy with Patients and Families*
*Project ECHO Autism in Transition Age Patients: Building a Community of Practice*	*Navigating Guardianship: Exploring the Use of a Group Education Model*
*Value Based Transition Payment Options and Considerations*	*Optimizing Billing Strategies related to Transition: Reimbursement for the Real Costs*

### 2.5 Evaluation and analysis of initiative

Notes from adult provider interviews were reviewed by 2 members of the workgroup to identify key concepts to be shared as lessons learned from successful adult provider models with broader initiative members. Data from the family survey were analyzed using counts and percentages of both yes/no questions and Likert scales. No statistical software was used.

In order to get feedback for the ongoing initiative, all participants were surveyed using QI satisfaction questions which were created by the initiative leadership. Both the steering (28 members) and taskforce (74 members) participants were queried on their experience of participating in the initiative itself, leading up to the summit. Everyone who agreed to be involved, regardless of whether they ultimately attended meetings or participated in any way, was included in this initiative participation survey. The survey included both Likert ratings (reported as counts and percentages) and open ended responses which were kept confidential. On a scale of 1–5 (1 =very unsatisfied, 2 =unsatisfied, 3 =neutral, 4 =satisfied, 5 =very satisfied) participants were asked to rate “What was your overall level of satisfaction with the process of the initiative over the last year?” On a scale of 1–5 (1 =No, not at all, 2 =No, not very much, 3 =neutral, 4 =Yes, somewhat, 5 =Yes, very good) participants were asked to rate “Do you feel your involvement was a good use of your time?” Two binary response questions were: “Do you feel you contributed toward the overall objectives of the initiative?” and “Would like to hear about continued opportunities to remain engaged and/or involved in the initiative?” Comment boxes were provided in the survey for participants to share additional details. Results of Likert scale responses were tallied as proportions of total responses and comments were examined for overarching themes by the initiative leadership.

All participants of the summit received a survey via email to complete as part of regular CME evaluation practice, which included quantitative and qualitative feedback that were provided to initiative leadership anonymously. Likert ratings described quantitative results. Responses to open-ended survey questions provided qualitative information including salient themes. Our institution considers QI projects to be exempt from IRB.

## 3 Results

### 3.1 Overall engagement with the initiative

A primary objective for the initiative was engaging solution-focused participation from across the state, defined in the charter as powerful collaboration with passionate stakeholders to identify best practices for improving the transition to adult healthcare for individuals with NDD. Consistent enthusiastic participation of stakeholders, with little attrition across the 18-month engagement process, demonstrated that outreach, convening and engagement was successful. Steering members consistently provided feedback during and between meetings that they found participation meaningful and they also continued to draw others into the work of the task forces in an effort to build the community of the initiative.

### 3.2 Results from key stakeholder adult provider interviews

Several themes emerged in looking at the successful adult practices. Facilitators identified included the presence of care coordination and the ability to provide accommodations via a flexible clinic structure. Barriers included difficulty with financing and inadequate provider training.

Care Coordination, defined as the co-creation (with the patient as developmentally appropriate, the family and/or caregiver) of a plan of care and its execution was noted to be a facilitator when done well. The successful programs provided specialized care coordination in support of integrated care management and were generally quite small, highly specialized “niche” practices that served patient populations with high severity needs, such as a clinic focused on adults with autism. Providers also noted that additional coordination was needed for patients who come regularly with staff members from group homes who often lack the full history or understanding of the patient to then share with the medical team.

Informants indicated that one important facilitator of successful care was their ability to provide accommodation for patient and family needs of this population in the clinical setting. For example, avoiding long wait times in crowded areas could lessen the disruption of having a loud, nonverbal patient with NDD who is behaviorally dysregulated in the waiting area.

A primary barrier identified involved financing. The programs we interviewed used a variety of funding, but it was clear that none survived on patient billing alone. Some clinicians were salaried and able to include the program within that role. Some used philanthropic funding, some inpatient billing, some a special funding mechanism provided by the state’s DDS program. From the interviews we learned that to scale or replicate, one would need to demonstrate cost savings, improved health, and/or better patient experience.

Another barrier involved confusion regarding the ideal location of the patients’ medical homes – whether it should be with the primary care provider (PCP) or specialty coordination programs. Several providers felt the PCP may be more important for adult patients in contrast to pediatrics where subspecialists may provide a medical home. Finally, all interviews noted the barrier of lack of knowledge in primary care providers and subspecialists such as adult psychiatrists and neurologists regarding the care of patients with NDD. There were only limited existing online resources from professional organizations and often these informants were responsible for teaching their peers via conferences, trainings, rounding, journal club, and direct consultation. However, this was not felt to be a scalable model.

### 3.3 Results of family/caregiver survey

The advocacy agency shared results from surveys received from 55 family caregivers of individuals with NDD, with the median age of those individuals reported as 22, ranging from age 3–54. Primary care was provided by a pediatrician (51 %), family medicine physician (21 %) or internal medicine physician (28 %). Ninety-three percent reported having a dentist, with 53 % of those as pediatric dentists, 38 % adult dentists and 8 % unsure. For those seeing an adult primary care internist, the mean age where transfer occurred was 21.2 years. For those who had not yet transferred care (n = 30), confidence was not high that it would happen successfully by age 26 (“not at all confident”, 26.7 %; “somewhat confident”, 36.7 %; “fairly confident”, 30 %; “very confident”, 6.7 %). However, for those that had transferred, the majority of respondents reported satisfaction in general (i.e. answering agree and strongly agree on all four satisfaction questions for primary care (n = 29)). For specialty care (n = 20) the majority of respondents agreed (44.4 %) or strongly agreed (16.7 %) that their medical needs are taken care of. However, there was less overall agreement on the remaining satisfaction questions. Please see Table 1.

### 3.4 Taskforce deliverables: collection/development of resources

The second part of the initiative goal involved identifying best practices for improving the transition to adult healthcare for individuals with NDD. The engaged stakeholders convened to create resources which could be shared broadly among providers in the state to avoid duplication of effort. For example, the Workforce Development taskforce compiled a list of available trainings and literature on trainings along with a spreadsheet of current requirements in areas of clinical training for physicians, subspecialists, and allied health providers. This included determining whether training on NDD of any kind is a component of expected training and licensing requirement for that clinical domain (e.g. internal medicine, neurology, etc.). The Optimizing Clinical Care taskforce did an environmental scan of current practice recommendations, drawing attention to a tool from our state Department of Developmental Services that had not been well known to participants. In addition, the taskforce created a workflow guideline related to assessing for guardianship needs in clinical practice. The Practice Management and Clinical Infrastructure taskforce created an “ideal state” process map to outline areas requiring attention and resources in daily practice. They also made recommendations for how to implement resources into clinical practice. Lastly, the Financing the Solution taskforce created several materials for “level-setting” for the participants, including educational trainings regarding financing and our state accountable care organization (ACO) process. They collated billing code tips and provided a framework to think about transition related to ACO needs. Beyond the task force deliverables, stakeholders were invited to present posters and 18 were accepted, with these resources added to the summit library.

### 3.5 Steering committee and taskforce engagement survey results

The four taskforces had assigned co-chairs, with 74 total participants. These groups also met monthly and worked together to create deliverables. After the Summit, all initiative participants, regardless of level of involvement in the steering committee or taskforces, were surveyed about overall experience of the entire initiative process. Twenty-seven people completed the survey, a 27 % response rate. When asked about overall satisfaction, all respondents (n = 27) were neutral to very satisfied, with no one reporting being very unsatisfied or unsatisfied (neutral, 5; satisfied, 13; very satisfied, 9).

All respondents (n = 27) indicated they felt neutral to very good about the use of their time, with no one reporting being that it was not at all or not very much (neutral, 2; somewhat, 13; very good, 12). When asked, “Do you feel you contributed toward the overall objectives of the initiative?”, 26 out of 27 participants responded yes. When asked, “Would like to hear about continued opportunities to remain engaged and/or involved in the initiative?” 24 of 26 respondents said yes.

The comment section in the survey allowed for additional qualitative information, including identification of barriers and facilitators of meaningful participation as the primary overarching themes. Within the theme of barriers, three sub-themes were identified. The first was related to “Technical Issues/Learning Zoom” and included comments such as: *“Zoom made it challenging to integrate the sub committees, so I felt like I didn't have the full picture of what would transpire at the conference”*. Another identified barrier sub-theme was “Lack of Time/Competing Demands”. Participants shared thoughts such as: *“My time this past year has been very stretched, especially with the pandemic”* and *“I missed some of the meetings towards the end of the process due to patient care needs”*. The final sub-theme regarding barriers was related to “Unclear Expectations” and included comments such as: *“I would have liked to have seen more clearly defined objectives for our task force”* and *“It seemed that the different groups were not talking to each other, and duplicate efforts were happening”*. Under the theme of facilitators, four sub-themes were identified. An important sub-theme was related to “Opportunities for Connection among the Dedicated”. Comments in this sub-theme reflected the high level of engagement and appreciation for various perspectives. For example, one participant commented: “*Working with an interdisciplinary group was very rewarding-while we shared the same passion the different perspectives on the challenges and present practices was enlightening; everyone seemed very committed to the summit and the NDD population”.* Another noted, *“I'm glad there was such engagement around this initiative from many different stakeholders”*. A second sub-theme, “Making an Effort”, aligned with our QI approach, highlighted the idea that any progress is positive. For example, a participant stated, *“Any effort to improve the transition to adult care for patients with NDD is appreciated”*. A third sub-theme regarding facilitators addressed the structure of the summit. “Unclear Expectations” were a sub-theme of barriers, but a mirroring sub-theme of “Structured Approach” appeared as a facilitator, with participants commenting: *“Regular meetings and discussion allowed us to move forward nicely”*. Lastly, a facilitator sub-theme of “What More” highlighted the sentiment that participants want to continue efforts and move progress forward with comments such as: *“Now that the basics are in place, time to move to measurable outcomes on the ground for youth, young adults, families, providers and payers.”*

### 3.6 Summit participant engagement survey results

**Fig. 2:**
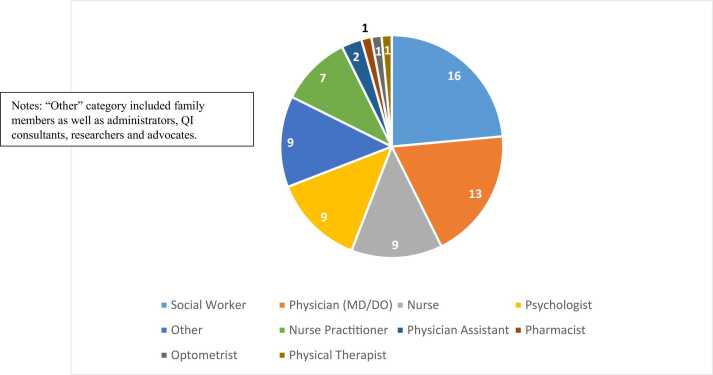
Summit Participant Demographics (N = 68).

Attendance at the summit of an even broader network of stakeholders represented positive engagement throughout the state as was our aim. There were 256 registrants for the summit, with diverse perspectives as outlined in Fig. 2. Since the summit offered continuing education credits for providers, evaluation was required for those requesting credit. All participants were encouraged to complete the survey. However, many people registered who did not require continuing education credit, especially family members, which may have lowered the response rate. Ultimately, sixty-five participants completed the post-summit survey (a 25 % response rate), with 100 % of them responding that all three objectives for the meeting were met. Respondents overwhelmingly (95 %) gave an overall rating of the summit as 4 (very good) or 5 (excellent) on a 5-point Likert scale. When asked how helpful the summit was for participants in setting transition related goals, 42 % found it “helpful” and 39 % “very helpful”. On a scale of “not at all” to “much better” related to having a better understanding of needs for the NDD population related to healthcare transition, 46 % of participant rated themselves “better” and 38 % “much better”. Rating themselves on a 5-point scale, 55 % rated themselves “better” and 38 % rated themselves “much better” in identifying areas for improvement to address gaps impacting healthcare transition. When asked to identify change ideas learned about at the summit, participants listed over 100 suggestions of possible changes to practice (see Table 3 for some examples).

**Table 3:** Example Change Ideas Identified at Summit.

Ideas to implement in clinical practice	Ideas to address macro (systems, process, education, etc) issues
Start transition period earlier	Get more involved in broader efforts for collaboration and advocacy with other stakeholders
Co-create the medical transition document with family & patient	Work directly with trainees on setting educational goals related to transition
Share DDS care guidelines	Create a policy for transition and identify patients in need of this plan
Be aware of the language I use when talking to others	Establish formal workflows between pediatrics and adult medicine to ease with transitions
Implement more interdisciplinary meetings across pediatric and adult providers	Develop a driver diagram with local stakeholders
Explore EPIC based tools	Research 2021 billing codes

## 4 Discussion

Given the growing population of adults with NDD^2^, ^3^, ^4^, ^5^ identified in national transition consensus statements as a “special population” vulnerable to poor outcomes ^6^, initiatives focused on improving the transition process for these individuals are crucial. The complexity of the systems and the diversity of the stakeholders involved requires deep engagement with interdisciplinary collaborators. Our initiative’s efforts to convene and engage diverse stakeholders across the state resulted in both meaningful networking connections and resource development. This represents one successful model to address the need for improved transition for individuals with NDD describing a statewide convening not otherwise seen in the literature.

“I want to see a big change with doctors' and patients' relationships.” This aspiration, expressed by a young adult patient panelist with multiple complex medical issues, helped set the tone for the inaugural summit of the *Massachusetts Initiative to Improve Healthcare Transition for Individuals with Neurodevelopmental Disabilities*. Transforming these relationships and fostering mutual engagement was a central objective for the entire initiative. Based on the feedback received, our model of using motivated participants coming from diverse perspectives working in taskforces on theme-based challenges succeeded in building relationships and developing resources collaboratively. What began as a workgroup of pediatric providers from one institution lamenting the challenges for themselves and their patients and families ended with a robust and diverse group of stakeholders engaged in a common purpose. To start, we were able to find some adult providers who had created model programs that were successfully caring for young adults with NDD. The identification of facilitators and barriers from those interviews set the stage for creation of the driver diagram which guided the effort. For example the financing taskforce engaged payer partners to explore some of these financing recommendations regarding value base payments and other billing opportunities. We were also able to share the perhaps surprising results from the family survey with key stakeholders throughout the initiative: that while families that had not gone through transition were not confident it would go well, the families that had transitioned reported overall satisfaction with adult providers and practices. Moreover, the structure provided within the initiative gave guidance for concrete action steps to leverage the strong sense of purpose into the creation of resources. With this approach, we were able to bring together all stakeholders with a shared goal of improving healthcare transition for those with NDD. The initial stakeholder engagement stage built a foundation. Developing a flexible and creative model was critical to our success.

Given the primary goal of the initiative was to engage in problem-solving with diverse stakeholders across the state, participation of over 100 people in regular meetings between steering and taskforces, and summit participation of over 200, represent strong outcomes. The experiences shared by initiative participants via survey indicated feelings that they contributed to the initiative and wanted to remain engaged, with a sub-theme of open responses identified specifically regarding “Opportunities for Connection among the Dedicated”.

Related to the second part of the initiative goal, participants were successful in identifying, creating and sharing resources to improve healthcare transition for those with NDD. Structured taskforce convening to identify and then create deliverables allowed for engagement that not only felt meaningful, but also ended with resources that were easily shared across many stakeholder settings. The main method of dissemination was the summit, allowing taskforce members to hear from one another, but also for the many other interested stakeholders who registered to learn from the efforts of initiative participants. The continuing education website platform was utilized as a resource library, from which participants could save materials.

Transition practice is generally approached using Quality Improvement (QI) methodology^14^, ^15^, ^16^, ^17^ and this initiative utilized a QI framework, including support from a QI consultant who encouraged participants in the use of relevant tools. Specifically, a driver diagram was created and refined both to anchor efforts and as a resource itself. It became the basis for not only resources but for process considerations. The overall methodology to consider drivers of change allowed for resource development and solution recommendations as was the other primary goal of the initiative. This included the deliverable development of the taskforces, and practical, small test-of-change ideas that were identified by participants of the summit as both feasible and achievable.

Identifying the prevalence of the target population was one of the major challenges of the initiative. Use of two different methodologies (population estimates of NDD prevalence^18^, approximately 150,000, and number of adults receiving services from the state department of Development Services, approximately 25,000) to estimate the total resulted in very disparate numbers with an over 6 fold difference. We also considered using medical records at various hospitals around the state but realized medical records do not always reliably identify those with NDD and there were difficulties sharing information across different medical record systems due to privacy and other data sharing issues. Furthermore, it was determined that use of billing codes would likely be inaccurate because they are done at point of service and the NDD code may not be used when a primary care practice sees the patient for gastroenteritis or an ear infection, for example. While ultimately, we were unable to determine exact numbers, stakeholders were brought together in the effort to problem solve a common denominator. There was strong consensus that a significant number of individuals likely have NDD, and therefore any systematic improvements to NDD care would be highly impactful to both patients and health systems.

The COVID-19 pandemic impacted our work significantly. Our plans for virtual taskforce collaboration were facilitated by participants’ rapidly increasing familiarity with virtual meetings. The shift to an online summit format from the planned in-person event allowed a broader depth of participation since attendees were able to access the summit remotely. This allowed busy clinicians to still attend parts of the summit if not the whole day. This would not have been accessible to them in an in-person format. This increased our overall number of summit participants and led to new ways of collaborating that can be utilized in the future.

### 4.1 Limitations

While we made significant efforts in participant recruitment, we were not able to consistently engage all stakeholder perspectives. For example, family partners were engaged but almost exclusively as parents, not with patient advocate involvement, and other perspectives may have been inadvertently missed. Our findings and experiences may not be representative of experiences from other parts of the United States. Virtual participation during the pandemic, on the whole, was a facilitator but technological and pandemic concerns were also notable challenges. While decreasing logistical challenges of in-person attendance, virtual convening may have inhibited some of the connections otherwise built with in-person engagement. Pandemic related clinical needs decreased clinicians’ ability to find time to participate in some work force groups and in the summit. In addition, there were some technical challenges during the summit itself that were barriers to full participation (e.g. lag time in accessing certain sessions for some participants). The summit website served as an important repository of the resources developed over the course of the initiative. However, as it was not built for this purpose, the platform provided the resources as they were presented at the Summit, and were not searchable as a library of resources. The library was also then available primarily for summit registrants, and not as an online resource that could be used easily by other interested stakeholders.

### 4.2 Future directions

Next steps for the initiative include annotation of the resource library, in collaboration with key stakeholders, to encourage dissemination and eventual sharing of resources. Students will be engaged to continue to build on the resources and create opportunities for sharing information. In addition to disseminating important resources, student engagement is a way to build a future workforce with knowledge of the topic. Further, based on the QI methodology, the driver diagram and identified solutions are available for testing by engaged teams. The next phase of the initiative includes establishment of an innovation collaborative to test out change ideas proposed by our broad stakeholder group.

## 5 Conclusion

A small group of providers had a goal to create a powerful collaboration with passionate stakeholders to identify best practices for improving the transition to adult healthcare for individuals with NDD. Through this initiative, a cohesive group was created to address the challenges and identify solutions and resources to be shared. Such learnings did and will continue to encourage stakeholders to advocate for a broad range of improvements across multiple settings. We demonstrated that through convening and engaging diverse and committed stakeholders and providing a structured program in which they could work, it is possible to begin to solve the major problem of healthcare transition for individuals with NDD. The lessons learned from engaging stakeholders across Massachusetts can be utilized by other groups wishing to create similar initiatives: 1) recruit diverse stakeholders including patient and family perspective to ensure robust understanding of the issues, 2) provide structure (e.g. taskforces) to carry out groundwork and create resources which encourages accountability, 3) highlight successes and use solution-focused approach to create momentum, and 4) convene to share lessons learned broadly which results in further engagement.

## Funding

This work was supported by a Special Interest Group Grant from the Mind Brain Behavior Institute at 10.13039/100006691Harvard Medical School; Bell Family Foundation.

## Ethical statement

Hereby, I, Susan Shanske, consciously assure that for the manuscript “A Statewide Initiative to Improve Healthcare Transition from Pediatric to Adult Care for Individuals with Neurodevelopmental Disabilities: Lessons Learned from Engaging Stakeholders” the following is fulfilled:

1) This material is the authors' own original work, which has not been previously published elsewhere. Early data was presented in poster format at the Health Care Research Consortium Symposium in October, 2022 in Houston, TX.

2) The paper is not currently being considered for publication elsewhere.

3) The paper reflects the authors' own research and analysis in a truthful and complete manner.

4) The paper properly credits the meaningful contributions of co-authors and co-researchers. No AI was used at any point in the manuscript creation.

5) The results are appropriately placed in the context of prior and existing research.

6) All sources used are properly disclosed (correct citation). Literally copying of text must be indicated as such by using quotation marks and giving proper reference.

7) All authors have been personally and actively involved in substantial work leading to the paper, and will take public responsibility for its content.

The violation of the Ethical Statement rules may result in severe consequences.

I agree with the above statements and declare that this submission follows the policies as outlined in the Guide for Authors and in the Ethical Statement.

## CRediT authorship contribution statement

**Lindsay MacAuley:** Writing – review & editing. **Susan Shanske:** Writing – original draft, Project administration, Methodology, Funding acquisition, Conceptualization. **Sarah Spence:** Writing – review & editing, Project administration, Methodology, Funding acquisition, Data curation, Conceptualization. **Rashid Kahn Tuba:** Writing – review & editing. **Lindsay Hunt:** Writing – review & editing, Supervision, Methodology. **Jamara Possemato:** Writing – review & editing, Project administration, Methodology, Data curation.

## Declaration of Competing Interest

The authors declare the following financial interests/personal relationships which may be considered as potential competing interests: Sarah Spence reports financial support was provided by Bell Family Fund. If there are other authors, they declare that they have no known competing financial interests or personal relationships that could have appeared to influence the work reported in this paper.

## Data Availability

No data was used for the research described in the article.
